# BK Channel Dysfunction in Diabetic Coronary Artery: Role of the E3 Ubiquitin Ligases

**DOI:** 10.3389/fphys.2020.00453

**Published:** 2020-05-29

**Authors:** Ling-ling Qian, Xiao-yu Liu, Zhi-ming Yu, Ru-xing Wang

**Affiliations:** Department of Cardiology, Wuxi People’s Hospital Affiliated to Nanjing Medical University, Wuxi, China

**Keywords:** diabetes mellitus, coronary artery, BK channel, ubiquitin–proteasome system, E3 ubiquitin ligase

## Abstract

Diabetic coronary arterial disease is a leading cause of morbidity and mortality in diabetic patients. The impaired function of large-conductance calcium-activated potassium channels (BK channels) is involved in diabetic coronary arterial disease. Many studies have indicated that the reduced BK channel expression in diabetic coronary artery is attributed to ubiquitin-mediated protein degradation by the ubiquitin–proteasome system. This review focuses on the influence and the mechanisms of BK channel regulation by E3 ubiquitin ligases in diabetic coronary arterial disease. Thus, BK channels regulated by E3 ubiquitin ligase may play a pivotal role in the coronary pathogenesis of diabetic mellitus and, as such, is a potentially attractive target for therapeutic intervention.

Diabetes mellitus represents a growing health problem worldwide (Hemmingsen et al., 2017). The injurious effects of diabetes mellitus include macrovascular and microvascular complications, such as brain, heart, kidney, retina, and other important organ damage (Shi and Vanhoutte, 2017). Diabetes mellitus has become one of the most important risk factors for the occurrence of coronary arterial disease (Komaru et al., 2020), leading to myocardial infarction, heart failure, and even sudden death (Junttila et al., 2018; Lorenzo-Almorós and Cepeda-Rodrigo, 2020). More and more studies in recent years have demonstrated that diabetes mellitus accelerates the progress of coronary heart disease and promotes the clinical manifestations of myocardial ischemia in advance (van den Heuvel et al., 2012). Thus, there is a critical need for research to elucidate the mechanisms involved in diabetic coronary arterial disease. However, the mechanisms of diabetes mellitus on the function of the coronary artery are not fully understood. An increasing body of evidence suggests that large-conductance calcium-activated potassium channels (BK channels) in coronary arterial smooth muscle cells (CASMCs) play an important role in diabetic coronary arterial disease (Wang et al., 2012a; Yi et al., 2014). In this review, we summarized the regulation of the coronary artery BK channels by the ubiquitin–proteasome system (UPS) in diabetes mellitus, especially E3 ubiquitin ligases, which may provide a new strategy for preventing and retarding the progress of coronary heart disease in patients with diabetes mellitus.

## Overview of the Ubiquitin–Proteasome System

**FIGURE 1:**
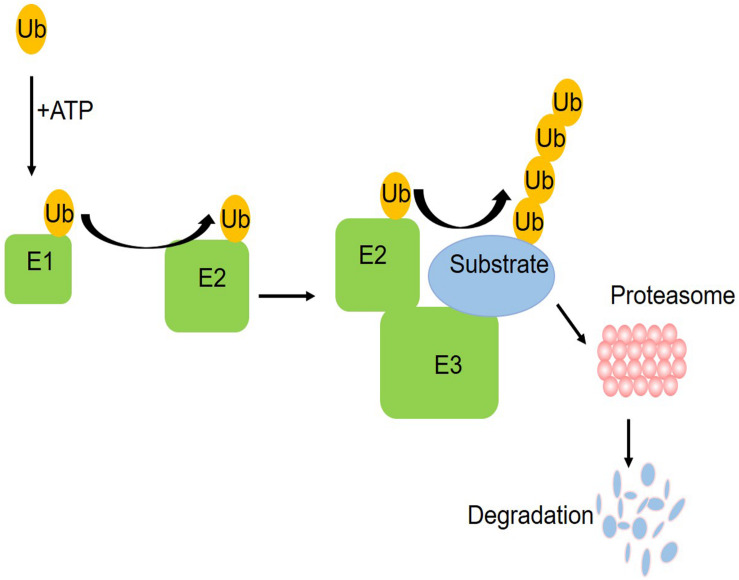
A schematic figure of the ubiquitin proteasome system. Initially, a single free ubiquitin was activated by E1 in an ATP-dependent manner and then the activated monoubiquitin molecule is transferred to a cysteine residue of the E2 enzyme, which is subsequently recruited into the E3 ligases. Working with a specific E3, E2 transferred one or more ubiquitin moieties sequentially to form ubiquitin chains to the substrate protein. Finally, the proteasome system can target the ubiquitinated substrate protein for degradation.

The ubiquitin–proteasome system (UPS) is one of the pathways for protein degradation in cells, where abnormal UPS function has been observed in different vascular diseases (Yu et al., 2017). The UPS includes ubiquitin, ubiquitin enzymes, proteasome, and its substrate proteins. The ubiquitin enzymes are divided into three categories: E1 ubiquitin activating enzyme, E2 ubiquitin binding enzyme, and E3 ubiquitin ligase (Barac et al., 2017). As shown in the schematic figure (Figure 1) of how the UPS works, initially, a single free ubiquitin was activated by E1 in an ATP-dependent manner and then the activated monoubiquitin molecule is transferred to a cysteine residue of the E2 enzyme, which is subsequently recruited into the E3 ligases. Working with a specific E3, E2 transfers one or more ubiquitin moieties sequentially to form ubiquitin chains to the substrate protein. Finally, the proteasome system can target the ubiquitinated substrate protein for degradation (Liu et al., 2015). In this process, a specific amino acid sequence and the phosphorylation domain of E3 help to identify the target proteins, ensuring that E2 will transfer the ubiquitin to the substrate. At present, only one E1 was found. There are more than 25 kinds of E2 and more than 1,000 kinds of E3. E3 ubiquitin ligases determine the specificity of the ubiquitination process. The ubiquitination of different substrate proteins depends on the specific E3. Accumulating evidence demonstrates the UPS alterations in diabetes mellitus (Shruthi et al., 2017; Reddy et al., 2018). These studies provided insights into the contribution of dysregulated UPS, especially diverse E3 ubiquitin ligases, to coronary vascular damage.

## BK Channel Dysfunction in Diabetic Coronary Artery

The BK channel, one of the most important potassium channels in CASMCs, plays an important role in regulating the vascular tone of the coronary artery. It has been reported that the BK channel currents account for about 65% of the total potassium channel currents in rat CASMCs. In structure, the BK channels mainly consist of two subunits: pore-forming α subunit and auxiliary β subunit, which form a tetramer (Ge et al., 2014). The α subunit is composed of seven transmembrane domains (S0–S6) (Miranda et al., 2018), including extracellular N terminals, voltage-sensitive domains, and cytoplasmic C ends with calcium and other regulatory molecule binding sites. There are four subtypes of β subunits (β1–β4) (Bhattarai et al., 2014), and mainly β1 subunit is in vascular smooth muscle cells. In addition to β subunits, recent studies have found that the auxiliary subunits of the BK channel also include the γ subunit and subunit LINGO1, which are rich in leucine repeat sequences (Zhang and Yan, 2014; Dudem et al., 2020). Auxiliary subunits are involved in regulating the voltage dependence and the calcium sensitivity of BK channels.

More and more studies have reported coronary BK channel dysfunctions in metabolic syndrome and different types of diabetes mellitus (Borbouse et al., 2009, 2010; Climent et al., 2017). The BK channels were depressed and coronary vasodilation to BK activator NS1619 was impaired in metabolic syndrome animal models (Burnham et al., 2006). In Zucker diabetic (ZDF) rats, the BK channels had impaired calcium sensitivity, with decreased maximal open probability, and a shortened mean open-time and prolonged mean closed-time durations (Lu et al., 2008). Compared with lean rats, the BK-β1 subunit expression was decreased, while the BK-α subunit remained in ZDF rats, as confirmed by immunoblotting analysis. In addition, the BK-β1 subunit-mediated activation by dehydrosoyasaponin-1 (DHS-1) was inhibited in CASMCs from ZDF rats (Lu et al., 2008). The BK-β1 subunit protein expression was decreased by 57.1% in high-fat-diet-induced type 2 diabetic (HFD) mice (Lu et al., 2017) and by 56.4% in db/db mice (Li et al., 2017), whereas the BK-β1 mRNA expressions remained normal.

In studies on streptozotocin (STZ)-induced type 1 diabetic models, similar changes of the BK channel were found as in type 2 diabetes mellitus (Lu et al., 2010; Zhang et al., 2010; Wang et al., 2012a, b; Tang et al., 2017). In STZ-induced diabetic rats, coronary BK-β1 subunit expression was also decreased and BK-α subunit expression remained in the coronary artery (Wang et al., 2012a, b; Tang et al., 2017; Zhang et al., 2010). Both open probability and current density were decreased in diabetic CASMCs, with coronary constriction to BK inhibitor iberiotoxin being markedly impaired (Wang et al., 2012a; Tang et al., 2017). The BK currents were decreased and the DHS-1-induced BK activation was impaired in CASMCs of STZ-induced diabetic rat (Zhang et al., 2010). Most studies reported that the BK-β1 subunit protein expression was decreased without a change of the BK-α subunit (Zhang et al., 2010; Wang et al., 2012a, b; Tang et al., 2017). One study (Lu et al., 2010) showed that the BK channel expression was unchanged in diabetic rats, although the function of the BK channel is damaged. However, they did not specify which subunit was detected in that report.

In addition to animal models, there is new evidence that the BK channel plays an important role in coronary dysfunction of human subjects with diabetes (Lu et al., 2019). Coronary arterioles were obtained from type 2 diabetic patients and non-diabetic subjects who had undergone a cardiopulmonary bypass procedure. BK channel activation by DHS-1 was diminished and BK channel-mediated vasodilation was impaired in diabetic coronary arterioles. The intrinsic properties of BK channels, such as sensitivity to Ca^2+^ and voltage, were downregulated in diabetic CASMCs. Differently from animals, the protein expressions of both the BK-α and the BK-β1 subunits were significantly downregulated in the coronary tissues of diabetic patients, without a change in the ratio of BK-α/BK-β1. In conclusion, coronary BK channel was dysregulated in diabetes.

The consequence of coronary BK channel dysfunction in diabetes has been reported. In db/db mice, the BK channel-dependent pathway was destroyed, resulting in the abnormal coupling of the TRPV1 channel-mediated coronary blood flow and cardiac metabolism (Guarini et al., 2012). It is also reported that the KCa channel activator can help to preserve the coronary blood flow of diabetic myocardium (Mishra et al., 2014). Impaired BK channel function in the coronary artery also exacerbated myocardial ischemia/reperfusion injury in STZ-induced diabetic mice (Lu et al., 2016). Thus, the BK channels might be a therapeutic target for diabetic coronary arterial diseases. However, the regulation mechanisms of diabetes mellitus on the coronary BK channels remain unclear.

## Regulation of BK Channels by E3 Ubiquitin Ligases in Diabetic Coronary Artery

The impaired BK channels lead to enhanced vasoconstriction in diabetic coronary arteries. Determining the mechanism by which E3 ubiquitin ligases affect the reduced BK channel expression can help to identify a novel strategy to protect against diabetic coronary arterial diseases. Several E3 ligases involved in BK channel ubiquitination have been reported, including F-box protein (FBXO) (Zhang et al., 2010), with-no-lysinekinase-4 (WNK4) (Wang et al., 2013), muscle RING finger protein 1 (MuRF1) (Yi et al., 2014), and CRL4A (CRBN). Among them, WNK4 affected the degradation of BK-α subunit, and overexpression of WNK4 decreased the BK-α subunit protein expression (Wang et al., 2013). Inactivation of CRL4A (CRBN) promoted the deubiquitinated BK channels released from the endoplasmic reticulum to the plasma membrane, leading to enhanced BK channel activity (Liu et al., 2014). Accumulated studies indicated that both FBXO and MuRF1 were important regulators of BK channels in diabetic CASMCs (Zhang et al., 2010; Yi et al., 2014).

### Regulation and Mechanisms of Coronary BK-β1 by FBXO

FBXOs are important components of the Skp1-Cullin-F-box-type ubiquitin ligase complex, usually as enzyme–substrate interaction sites (Kipreos and Pagano, 2000). The expression of the FBXO protein is controlled by the forkhead box O family transcription factor (FOXO), and the activity of FOXO is negatively regulated by Akt. Akt promotes the phosphorylation of FOXO at T32, S253, and S315. The phosphorylated FOXO gets out of the nucleus, losing transcriptional function (Sandri et al., 2004). FBXO-9 and FBXO-32 are two of the FBXO subtypes specifically expressed in muscle cells (Gomes et al., 2001; Li et al., 2007). FBXO-32 can bind to the PDZ binding motifs of substrate proteins (Gomes et al., 2001), and the BK-β1 subunit has such a PDZ binding motif (Zhang et al., 2010).

The BK channel current density was reduced and DHS-1-mediated channel activation was impaired in freshly isolated CASMCs of STZ-induced diabetic rats (Wang et al., 2012a). Zhang et al. reported that the BK-β1 subunit expression was downregulated and the BK-β1 subunit ubiquitination was enhanced both in the aorta vessels of STZ-induced diabetic rats and high-glucose cultured human CASMCs (Zhang et al., 2010), accompanied by the significantly increased expression of FBXO-9 and FBXO-32. The BK-β1 subunit expression was 1.65 times upregulated by FBXO-9 small interfering RNA but suppressed by the FBXO agonist doxorubicin in human CASMCs. Meanwhile, the protease inhibitor MG-132 can block the effect of doxorubicin (a FBXO activator) on the expression of the BK-β1 subunit, indicating that FBXO is involved in the regulation of BK-β1 subunit protein degradation *via* UPS. In HEK293 cells, co-transfected with human BK-β1 subunit gene (Flag-hSlo-β1) and FBXO-32, the BK-β1 subunit ubiquitination was enhanced and the BK-β1 subunit expression was reduced, but in HEK293 cell transfection of human BK-β1 subunit gene mutation without the PDZ binding motif, the BK-β1 subunit protein level was not changed. These results confirmed that FBXO interacted with BK channels through the PDZ binding motif of BK-β1 subunit and facilitated BK-β1 subunit protein ubiquitination and degradation. In diabetes mellitus, the expression of FBXO was influenced by the decreased phosphorylation of FOXO-3a. Decreased Akt and FOXO-3a phosphorylation, enhanced FBXO-32 expression, increased BK-β1 subunit degradation through ubiquitination, and suppressed BK channels expression in the vascular tissues of STZ-induced rats and high-glucose cultured human CASMCs were mimicked by Akt inhibition with LY294002 in human CASMCs, which suggested that the phosphorylation of Akt is associated with the ubiquitination of the BK-β1 subunit by FBXO.

Further studies were performed in the vascular tissue of STZ-induced diabetic mice and high-glucose cultured human CASMCs to find the upstream signaling of FBXO. Decreased BK-β1 subunit protein, accompanied by increased FBXO-32 protein expression, decreased FOXO-3a phosphorylation, and decreased Akt phosphorylation were found (Lu et al., 2012), consistent with the treatment with phosphatidylinositide 3-kinases (PI3K) inhibitor LY294002. Thus, the PI3K/Akt signaling regulated the BK-β1 subunit expression *via* FOXO-3a/FBXO-32 in diabetes mellitus. As reported, PI3K/Akt signaling is suppressed by protein kinase C (PKC) and activated by insulin. In diabetes mellitus, the PKC activity is enhanced and insulin receptor is diminished. These might lead to the upregulation of FBXO transcription and impairment of BK channel functions *via* increased BK-β1 ubiquitination by FBXO.

In fact, the increased expression of PKCβ stimulated the NADP oxidases activity to overproduce reactive oxygen species (ROS), which inhibits the PI3K/Akt pathway in diabetic condition. Hydrogen peroxide incubation increased the expression of FBXO-32 and decreased the expression of the BK-β1 subunit in human CASMCs (Lu et al., 2012). Hence, abnormal ROS promoted the Akt/FOXO-3a/FBXO-32-dependent regulation of BK channel degradation in diabetes mellitus. PKC/ROS activation was also attributed to caveolae-1 upregulation-mediated angiotensin II type 1 receptor signaling in STZ-induced diabetic rats. In addition, PKC inhibitor ruboxistaurin or peroxisome proliferator-activated receptor agonist GW501516 can reverse the effect of high-glucose-induced Akt/FOXO-3a/FBXO-32 signaling pathway and increase the expression of the BK-β1 subunit protein in high-glucose cultured human CASMCs. However, these two reagents showed different effects on the setting of normal- or high-glucose culture. The effects of ruboxistaurin on enhancing the BK-β1 expression were more potent in human CASMCs cultured with normal glucose than with a high one. This may be due to the complexity of the PKCβ/ROS signaling cascades and there may be other downstream pathways of ROS signaling involved in regulating BK-β1 degradation in diabetic coronary arterial disease. GW501516 reduced the BK-β1 expression in normal-glucose cultured cells through Akt inhibition, which is contrary in a high-glucose culture. The reason may be the changes in intracellular redox homeostasis and signaling transduction in different glucose levels. Further studies showed that the oral administration of ruboxistaurin or GW501516 preserved BK-β1 expression and BK channel activator NS-1619-induced coronary vasodilation in STZ-induced diabetic mice.

To summarize these studies, the decreased expression of the BK-β1 subunit (Zhang et al., 2010; Wang et al., 2012a; Tang et al., 2017), increased FBXO-9 and FBXO-32 (Zhang et al., 2010), and decreased phosphorylated Akt and FOXO-3a (Zhang et al., 2010; Lu et al., 2012) were observed in the vascular tissues of diabetic animals. The same protein expression changes were also confirmed in human CASMCs with high-glucose culture (Zhang et al., 2010; Lu et al., 2012); FBXO-9 small interfering RNA, FBXO agonists, Akt inhibitors, etc., were added in cell culture respectively to verify the role of these related molecules in this pathway (Zhang et al., 2010; Lu et al., 2012). After that, PKC (upstream of Akt) inhibitors were given to diabetic animals and vascular tissues (Lu et al., 2012) were detected to further confirm the role of Akt/FOXO-3a/FBXO signaling pathway in regulating the ubiquitination of BK channel. So, these data from different systems are closely related. Thus, these observations may help to develop new strategies for the treatment of diabetic coronary arterial diseases.

### Regulation and Mechanisms of Coronary BK-β1 by MuRF1

The muscle ring finger protein family has three members, including MuRF1, MuRF2, and MuRF3. They are a group of muscle-specific E3 ligases. These three subtypes are abundant in muscle tissue, and they contain four important domains: a ring finger domain, a conserved region of the MuRF family, a “B-box” domain, and multiple coiled-coil domains (Mrosek et al., 2008). MuRF1 has been proved to be involved in the regulation of various cardiovascular diseases (Willis et al., 2007), including myocardial hypertrophy, myocardial ischemia, and myocarditis. In recent years, it is reported that MuRF1 has been involved in the regulation of vascular function (Yi et al., 2014).

In STZ-induced diabetic mice, BK channel current was decreased in freshly isolated CASMCs, accompanied by increased MuRF1 protein, enhanced BK ubiquitination level, and degradation of the BK-β1 subunit in aorta tissues (Yi et al., 2014). Similar effects were found in high-glucose cultured human CASMCs, in which downregulated MuRF1 expression by small interfering RNA significantly increased the BK-β1 subunit protein expression, while the overexpression of MuRF1 by adenovirus transfection decreased the expression of the BK-β1 subunit. A pulldown assay demonstrated that the N terminal of the BK-β1 subunit interacted with the coiled-coil domain of MuRF1. The relaxation to BK channel activator NS1619 was attenuated in the coronary artery with adenovirus-transfected MuRF1. Incubation of MG132 (a proteasome inhibitor) restored the effect of the overexpression of MuRF1. Thus, MuRF1 reduced BK channel activation and impaired vasorelaxation *via* MuRF1-dependent BK-β1 subunit ubiquitination and degradation.

MuRF1 mRNA transcription and protein expression in diabetic vascular tissue were upregulated by the activation of nuclear factor kappa B (NF-κB) (Yi et al., 2014) and nuclear factor E2-related factor 2 (Nrf2) (Li et al., 2017). The Nrf2 signal regulates cellular redox status. In the kidney, myocardium, and vascular smooth muscle cells, the activation of Nrf2 protects against the apoptosis induced by oxidative stress and plays a protective role in diabetes mellitus (Li et al., 2012). In the arterial tissue of diabetic db/db mice, the BK-β1 subunit protein was reduced, accompanied by increased MuRF1 and decreased Nrf2 protein expression. These effects were mimicked by high-glucose culture or downregulation of Nrf2 in human CASMCs. Conversely, the overexpression of Nrf2 by adenovirus transfection or agonist dimethyl fumarate (DMF) can suppress MuRF1 and enhance BK-β1 subunit expression (Li et al., 2017).

Reduction of the BK-β1 protein in the coronary arteries of HFD mice was also observed to be similar in type 1 diabetic mice, mainly due to accelerated proteolysis through the UPS. The protein expression of Nrf2 and BK-β1 was markedly downregulated while those of NF-κB and MuRF1 were significantly upregulated in HFD mice (Lu et al., 2017). Nrf2 was the upstream signal of NF-κB. After transfection of Nrf2 in human CASMCs, the expression of NF-kappa B/p50, NF-κB/p65, and p-NF-κB/p65 was decreased but the BK-β1 subunit expression was enhanced. Knockdown of Nrf2 resulted in opposite changes of these proteins. By treating with Nrf2 agonist, DMF, activation of Nrf2 preserved the BK-β1 subunit expression that protected BK channel functions and BK channel-mediated coronary dilation in high-fat-diet-induced diabetic mice. The regulation of vascular BK-β1 protein expression in HFD mice by Nrf2 was mediated through NF-κB/MuRF1-dependent proteolysis. Hence, Nrf2 is a novel regulator of BK channel functions with therapeutic implications in diabetic coronary arterial disease. However, it was also found that Nrf2 overexpression upregulated the BK-β1 mRNA expression in cultured human CASMCs. The researchers speculate that the possible mechanism is that the BK-β1 gene contains several consensus sequences of Nrf2-binding motifs in its promoter. It suggests that Nrf2 not only regulates BK channel ubiquitination through MuRF1-independent pathway but may also directly increase the BK-β1 mRNA transcription or indirectly regulate other transcriptional factors responsible for BK-β1 transcription. As the BK channel plays an important role in the regulation of coronary blood flow (Guarini et al., 2012; Mishra et al., 2014) and myocardial ischemia/reperfusion injury (Lu et al., 2016) in diabetes, the coronary arteries of STZ-induced SD rats were investigated in several studies (Lu et al., 2010; Wang et al., 2012a; Tang et al., 2017), including the diameter at baseline and after IBTX application (Wang et al., 2012a) as well as the responses to a single concentration of angiotensin II, IBTX, NS1619 (Lu et al., 2010; Tang et al., 2017), etc. However, after intervention by siRNA or drugs [including PKC and PPAR receptor inhibitors (Lu et al., 2012), Ad-MuRF1 (Yi et al., 2014), Nrf2 agonists (Lu et al., 2017), etc.] in intact vessel or animal model, most of the reports studied the relaxation response of coronary vessels to different concentrations of NS1619, as in a single experimental series. The vascular effects to shear stress and NS1619 were both observed in only one study in db/db mice (Li et al., 2017). Thus, the evidence in intact vessels may be a little weak. More literatures are needed to find out the role of E3 ubiquitin enzymes, such as FBXO and MuRF1, in the regulation of vascular function through the BK channel, including the vascular response to vasoconstrictors and vasodilators, the baseline diameter or tone, the reactivity to IBTX, and even coronary blood flow. In these studies, NS1619 has been used to explore the role of BK channel in the regulation of vascular function (Lu et al., 2010, 2012, 2017; Yi et al., 2014; Li et al., 2017). NS1619 was considered as the activator of the BK channel and played the vasodilation role through BK channel activation. However, it was also reported that NS1619 can also cause vasodilation by affecting multiple complementary pathways, including the stimulation of NO production, regulating other K^+^ channels and L-type Ca^2+^ channels (McCullough et al., 2014), i.e., NS1619 may have a non-specific activation effect on BK channel activation. Besides that, more and more studies have reported that NS1619 is the activator of the BK-α subunit (Reed et al., 2019; Du et al., 2020). Therefore, the single experiment of vascular response to NS1619 is not enough to prove the effect of the BK channel. IBTX, a specific inhibitor of BK channel, or other activators of BK channel should be used in further studies.

In summary, FBXO and MuRF1 are two critical E3 ubiquitin ligases involved in coronary BK-β1 ubiquitination and degradation in diabetes. The PDZ motif (interacted with FBXO) of BK-β1 is located at the extracellular loop (Zhang et al., 2010), while the N-terminal (interacted with MuRF1) of BK-β1 is intracellular (Yi et al., 2014). An increase of both MuRF1 and atrogin-1 expression in diabetic CASMCs may synergistically accelerate BK-β1 protein degradation and exacerbate BK channel-mediated coronary arterial dysfunction in diabetes. Among these studies that we reviewed, a lot of data about BK channel were obtained from different systems. HEK293 cells were used as an artificial expression system to study the specific role of a subunit or a site (Zhang et al., 2010). Human CASMCs were cultured to explore the change of protein level and freshly isolated CASMCs were used to record the BK channel currents (Lu et al., 2010, 2012, 2017; Zhang et al., 2010; Yi et al., 2014; Li et al., 2017) as the BK currents can hardly be recorded in cultured CASMCs. In most of the study data that we reviewed, the changes of the BK channel and other proteins were observed first in different vascular tissues of diabetic animal models. Then, human coronary artery smooth muscle cells were cultured with high glucose to mimic diabetes; similar changes were found, and further mechanisms and the key possible related molecules were verified in cultured cells. After finding the key possible related molecules, their inhibitors or agonists were used in a diabetic animal model to get more data from vascular tissues. The data obtained from these different systems are verified from multiple perspectives to reach a consistent conclusion. Besides that, both type 1 (Lu et al., 2010; Zhang et al., 2010; Wang et al., 2012a, b; Yi et al., 2014; Tang et al., 2017) and type 2 diabetic (Li et al., 2017; Lu et al., 2017) models were established to study the role of the coronary BK channel. These results are consistent and suggest that the BK channel dysfunction in diabetes is related to the increased degradation of the BK channel *via* ubiquitination of the BK-β1 subunit. Therefore, we speculated that the impairment of the BK channel in diabetic coronary artery might be more related to hyperglycemia than to the insulin level. The molecular mechanism verified by high-glucose culture in human CASMCs was consistent with the diabetic models.

The data of these reports are very detailed, but there are still some flaws. Due to the limitation of the amount of coronary artery tissues in rodents, some western blot experiments used the aortic tissue instead of the coronary arteries to detect BK channel protein expression, although the BK channel currents and vascular function were detected by using coronary artery tissues (Lu et al., 2010, 2012, 2017; Zhang et al., 2010; Yi et al., 2014; Li et al., 2017). Although it may not affect the results, the tissues came from different vascular beds. In the Western blot experiment, full-length blots were not shown and multi-antibodies of BK-β1 were used in different studies. Some studies used the antibody of BK-β1 from companies (Zhang et al., 2010; Wang et al., 2012a; Tang et al., 2017) which can provide a specific antigen to verify the specificity of the antibody, while others used custom-made antibodies (Lu et al., 2010, 2012, 2017; Yi et al., 2014; Li et al., 2017), but all of them did not show the specificity of the antibodies, which may affect the reliability of the data.

## Conclusion

**FIGURE 2:**
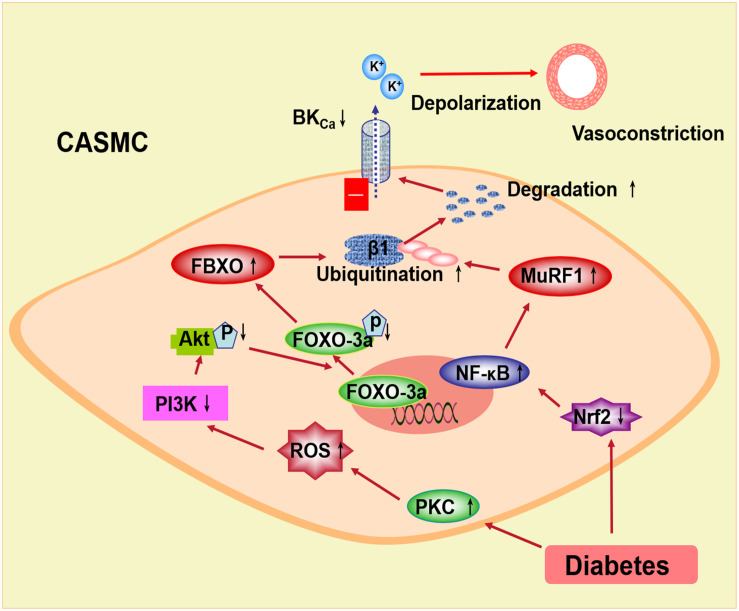
Proposed signaling pathways underlying the BK channel downregulation through E3 ubiquitin ligases in coronary smooth muscle cells. In diabetes, increased PKC stimulation overproduced ROS, which inhibits the PI3k/Akt pathway. Decreased Akt phosphorylation suppresses FOXO-3a and then upregulates FBXO expression. Nrf2 is downregulated in diabetic coronary smooth muscle cells. Decreased Nrf2 upregulates NF-κB and MuRF1. FBXO and MuRF1 are muscle-specific E3 ubiquitin ligases, which enhance the ubiquitination and the degradation of the BK β1-subunit. The coronary vasoconstriction in diabetes is increased due to the impaired BK channel expression and function. PKC, protein kinase C; ROS, reactive oxygen species; PI3k, phosphatidylinositide 3-kinases; FOXO-3a, the forkhead box O family transcription factor-3a; FBXO, F-box protein; Nrf2, nuclear factor E2-related factor 2; NF-κB, nuclear factor kappa B; MuRF1, muscle RING finger protein 1.

In conclusion, the BK channel function is impaired and the expression of the BK-β1 subunit is decreased in diabetes mellitus, which may be related to the ubiquitination and the degradation of the BK-β1 subunit through E3 ubiquitin ligases (Figure 2). In diabetic vascular tissue, ROS is increased and Akt phosphorylation is suppressed, leading to inhibiting FOXO-3a phosphorylation and enhancing FBXO-32 protein expression. Also, the expression of Nrf2 is decreased in diabetes mellitus, resulting in reduced NF-κB activation and upregulated MuRF1 protein. FBXO and MuRF1 are two muscle-specific E3 ligases, which enhance the ubiquitination of the BK-β1 subunit. The expression and the function of the BK channels are impaired and considered as the cause of increased BK-β1 subunit ubiquitination. The protein expression of the coronary BK-β1 subunit and coronary vascular function can be preserved in diabetes by the intervention of the E3 ligase-related pathway, such as the PKC inhibitor or the Nrf2 agonist. Thus, activation of Akt or Nrf2 may inhibit the ubiquitination of the BK channels through the FBXO- or MuRF1-dependent pathway and protect the diabetic coronary arteries, which may provide a new strategy for the treatment of diabetic coronary arterial diseases.

## Author Contributions

LQ and XL wrote the manuscript. ZY and RW edited the manuscript. All the authors read and approved the final manuscript.

## Conflict of Interest

The authors declare that the research was conducted in the absence of any commercial or financial relationships that could be construed as a potential conflict of interest.

## Abbreviations

BK channels: large-conductance calcium-activated potassium channels
CASMCs: coronary arterial smooth muscle cells
DHS-1: dehydrosoyasaponin-1
FBXO: F-box protein
FOXO: the forkhead box O family transcription factor
HFD mice: high-fat-diet-induced type 2 diabetic mice
MuRF1: muscle RING finger protein 1
NF- κ B: nuclear factor kappa B
Nrf2: nuclear factor E2-related factor 2
PI3K: phosphatidylinositide 3-kinases
PKC: protein kinase C
ROS: reactive oxygen species
UPS: ubiquitin proteasome system
WNK4: with-no-lysinekinase-4
ZDF rats: Zucker diabetic rats.
